# Medical Bacteriology: a Practical Approach

**DOI:** 10.3201/eid1105.050224

**Published:** 2005-05

**Authors:** Robbin S. Weyant

**Affiliations:** * Centers for Disease Control and Prevention, Atlanta, Georgia, USA

**Keywords:** Medical Bacteriology, Clinical Bacteriology, Bacteriological Methods

Medical Bacteriology is a multicontributor work with chapters provided by various expert medical microbiologists from the United Kingdom. The information is organized into 4 basic areas totaling 12 chapters: 6 chapters covering the analysis of various types of patient specimens; 2 chapters on antimicrobial analysis, susceptibility testing, and direct assay in patient specimens; 2 chapters on laboratory management issues, including information technology, quality control, and quality assurance; and 2 chapters on the role of the laboratory in hospital infection control programs and in the support of epidemiologic investigations. Distributed throughout the text are 81 individual testing protocols, which can be found either by referring to a table at the front of the book or by searching the index. Also included are 4 appendixes.

Multiple options exist for presenting the fundamentals of medical bacteriology. Some texts have used a disease-based approach. Other have used an organism-based approach. Medical Bacteriology takes a specimen-based approach: bacterial diseases and their causative agents are addressed through the proper collection, processing, and analysis of clinical specimens. From a laboratory perspective, the specimen-based approach has substantial advantages. In this text, the critical importance of proper collection and transport of specimens is clearly communicated for each specimen type, and appropriate protocols are provided. In addition, an in-depth discussion is presented on the proper interpretation of cultures and other laboratory findings for each specimen type. For example, the chapter on urine bacteriology contains not only guidelines for interpreting various types of urine cultures, but also the relevance of other routine urinalysis findings to infections of the urinary tract. One disadvantage of the specimen-based approach is that organism identification protocols may appear in multiple chapters.

The chapters devoted to antimicrobial issues provide basic testing protocols and valuable insights on the selection of appropriate agents for routine testing and reporting. The relative advantages and disadvantages of diffusion versus dilution methods are clearly described, and readers from the United States may find some of the alternatives to the standard Kirby-Bauer procedure to be of interest.

The chapters devoted to laboratory management provide excellent insights on the rapidly evolving field of laboratory information technology. Laboratorians should find the comparison of stand-alone "legacy" systems with more integrated hospital-wide and health system–wide designs useful in making decisions on laboratory information management.

The chapters on infection control and epidemiology provide overview of the technical and administrative issues encountered by the laboratory. The organization and function of hospital infection control committees are discussed, and guidance for performing basic cohort and case-control studies is presented.

Emphasis is placed on the processing and analysis of specimens with well-established protocols and materials. Bacterial identification and characterization protocols are based on techniques that have been in general use for many years. Readers interested in newer approaches and in analyses of some of the more exotic bacterial zoonoses are provided with appropriate references.

The book flows smoothly from chapter to chapter. Similar material appearing in various chapters is appropriately cross-referenced. The text is clearly written, with jargon and acronyms kept to a minimum. At 409 pages, including the appendixes and index, this book can be easily read in a few sittings, and readers, including students, technologists, laboratory supervisors, and senior scientists, should find it to be a useful reference.

